# Flexible Gait Sensing and Machine Learning Recognition Based on Phase-Separated PVDF-HFP Films

**DOI:** 10.3390/s26165270

**Published:** 2026-08-20

**Authors:** Huimin Liang, Qi Shao, Fuhao Wu, Yibo Xiong, Wenwu Wang, Hongbin Su, Xiyao Huang, Zilu Hu, Yixin Wang, Liang He

**Affiliations:** 1 School of Mechanical Engineering, State Key Laboratory of Intelligent Construction and Healthy Operation and Maintenance of Deep Underground Engineering, Sichuan University, Chengdu 610065, China; liang_huimin@stu.scu.edu.cn (H.L.); shaoqi@stu.scu.edu.cn (Q.S.); wufuhao@stu.scu.edu.cn (F.W.); 2023223020040@stu.scu.edu.cn (Y.X.); wangwenwu@stu.scu.edu.cn (W.W.); m15095542960@163.com (H.S.); huangxiyao111@stu.scu.edu.cn (X.H.); huzilu@stu.scu.edu.cn (Z.H.); 2 School of Mechatronical Engineering, Beijing Institute of Technology, Beijing 100081, China; 3120235452@bit.edu.cn

**Keywords:** piezoelectric sensor, machine learning, gait recognition, poly(vinylidene fluoride-co-hexafluoropropylene)

## Abstract

Flexible wearable piezoelectric sensors have attracted increasing attention in human motion monitoring and motion classification applications due to their self-powered sensing capability and rapid response. In this work, poly(vinylidene fluoride-co-hexafluoropropylene) (PVDF-HFP) flexible piezoelectric films were fabricated using a phase separation method with different loading masses of PVDF-HFP to regulate the crystal structure and output signal characteristics of the films. X-ray diffraction and Fourier-transform infrared spectroscopy analyses demonstrated that an appropriate mass of PVDF-HFP promoted the formation of polar β-phase crystals, and the optimized film exhibited a β-phase content of 86.81%. The prepared films generated stable and distinguishable response signals under different gait conditions, indicating high potential for flexible motion sensing. Furthermore, machine learning-assisted motion classification was preliminarily performed based on the acquired sensing signals, achieving an accuracy above 90%. This work demonstrates the potential of phase-separated PVDF-HFP films for flexible gait sensing and wearable motion recognition applications.

## 1. Introduction

Flexible wearable electronics have attracted increasing attention in the fields of healthcare monitoring [1], human–machine interaction [2,3], and intelligent motion analysis in recent years. Among various human motion behaviors, gait contains abundant physiological and motion-related information. Through the monitoring and analysis of gait signals, applications such as motion evaluation [4], rehabilitation assistance, and elderly health monitoring can be realized. Therefore, flexible gait sensors capable of real-time dynamic monitoring have gradually become a research focus.

Among various flexible sensing technologies [5], piezoelectric sensors are considered highly suitable for human motion signal detection due to their self-powered characteristics, fast response, and high sensitivity to dynamic pressure. Piezoelectric materials can directly convert mechanical stimuli generated during human motion into electrical signals, enabling real-time perception of different motion states and providing a promising foundation for flexible wearable sensing applications.

Poly(vinylidene fluoride-co-hexafluoropropylene) (PVDF-HFP) has demonstrated broad application potential in flexible piezoelectric sensing owing to its excellent flexibility, biocompatibility, and processability [6,7]. The piezoelectric response of PVDF-HFP is highly dependent on the content of polar β-phase crystals [8,9]. Therefore, effectively promoting β-phase formation is essential for improving its flexible sensing performance. At present, methods such as mechanical stretching, electric field poling, and thermal treatment are commonly employed to enhance β-phase formation in PVDF-based materials [10,11]. Although these methods can improve the piezoelectric response to a certain extent, they usually require multistep processing or additional energy input, resulting in complicated fabrication procedures, relatively high energy consumption, and limitations in large-area flexible fabrication [12,13]. Therefore, developing a simple and efficient strategy suitable for the rapid fabrication of flexible films is of great significance for the practical application of wearable piezoelectric sensors. In comparison, phase separation methods [14] have been widely applied in the fabrication of PVDF-based flexible piezoelectric films in recent years because of their simple processing, rapid film formation, and ability to facilitate β-phase formation.

However, few studies have explored how the dosage of PVDF-HFP in the phase separation process affects the crystal structure and flexible sensing performance of PVDF-HFP thin films. Therefore, in this work, PVDF-HFP flexible piezoelectric films with different loading masses of PVDF-HFP were prepared using a spray-assisted phase separation method, and the effects of the mass of PVDF-HFP on β-phase formation and electrical sensing behavior were systematically investigated. The optimized film was further applied to wearable gait monitoring under different human motion states, and machine learning-based motion recognition was subsequently performed using multiple classification models. This work demonstrates a simple strategy for constructing flexible wearable sensors with intelligent motion recognition capability.

## 2. Materials and Methods

### 2.1. Preparation of PVDF-HFP Sensor

**Figure 1:**
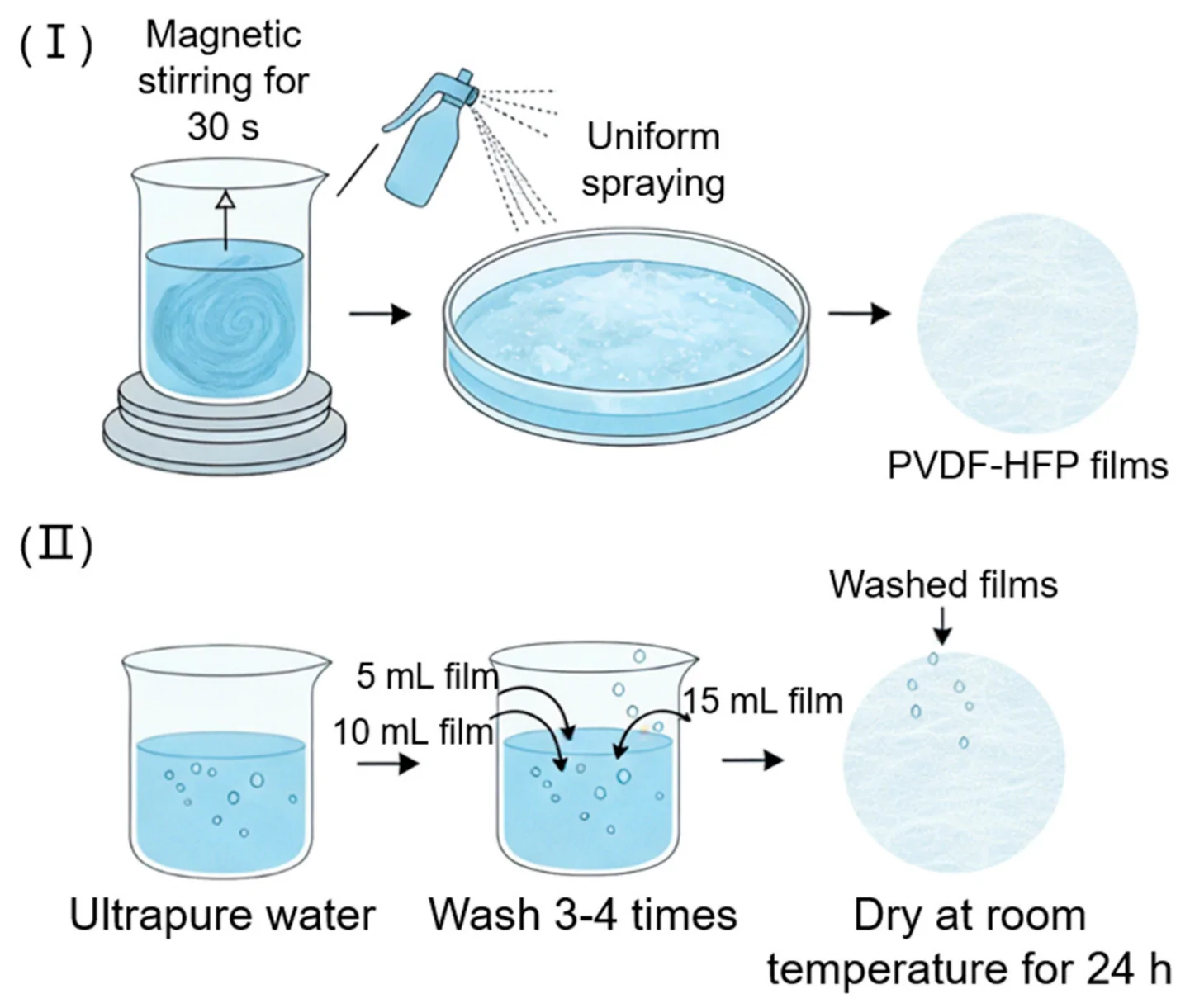
Schematic illustration of the fabrication process of phase-separated PVDF-HFP films: (**I**) preparation of the PVDF-HFP precursor solution and spray-assisted phase separation on the ice substrate; (**II**) washing and drying procedures of the fabricated films.

The preparation process of the PVDF-HFP films is illustrated in Figure 1. In this study, the films were fabricated using a phase separation method. For the preparation of the PVDF-HFP precursor solution, PVDF-HFP pellets (average *M*_w_ = 600,000 g/mol, Solvay, Lyon, France) were dissolved in *N*,*N*-dimethylacetamide (DMAc, purity ≥ 99.0%, Macklin, Shanghai, China) at a concentration of 5 wt.%. The mixture was magnetically stirred at 400 rpm for 1 h until a homogeneous and transparent solution was obtained, followed by storage in a refrigerator to maintain a low-temperature state.

During the phase separation process, ice was employed as the substrate, which can suppress polymer chain relaxation caused by Brownian motion and facilitate the stabilization of β-phase formation in PVDF-HFP films. The detailed preparation procedure is described as follows:

(I) Ultrapure water was frozen in a Petri dish (*d* = 9 cm) for 24 h to obtain an ice substrate. To investigate the influence of precursor loading on the phase separation behavior of PVDF-HFP films, different volumes of PVDF-HFP precursor solution (5 mL, 10 mL, and 15 mL) were separately loaded into a spray gun and vertically sprayed onto the ice substrate. The corresponding PVDF-HFP loadings were 0.25, 0.50, and 0.75 g, respectively. After standing at room temperature until the ice completely melted, the phase-separated films were peeled off the substrate.

(II) The obtained films were rinsed with ultrapure water 3–4 times to remove residual solvent and then dried at room temperature for 24 h.

For the fabrication of the piezoelectric sensor, copper foil was attached to both sides of the PVDF-HFP film (2 cm × 2 cm) as electrodes, and conductive wires were connected between the electrodes and the film for piezoelectric signal output. The device was subsequently encapsulated using polyimide film to reduce environmental interference during signal acquisition. Finally, the output wires were connected to a printed circuit board (PCB) for device-level signal denoising, completing the fabrication of the flexible piezoelectric sensor.

### 2.2. Characterizations

PVDF-HFP generally exhibits two dominant crystalline phases, namely the nonpolar α-phase and the polar β-phase, in which the β-phase is mainly responsible for the piezoelectric response of the material. To investigate the crystal structure of the prepared PVDF-HFP piezoelectric films, X-ray diffraction (XRD) analysis was performed using an XRD-6100 diffractometer (Shimadzu Corporation, Kyoto, Japan) with Cu-Kα radiation at a scanning rate of 5 °/min.

Based on the XRD patterns, Raman spectroscopy and Fourier-transform infrared spectroscopy (FTIR) measurements were further conducted using a Nicolet iS50 spectrometer (Thermo Scientific, Waltham, MA, USA) to quantitatively evaluate the β-phase content of the samples. In the FTIR spectra, characteristic absorption bands located at 615, 765, and 975 cm^−1^ correspond to the α-phase, while the absorption band at 840 cm^−1^ is attributed to the β-phase.

The relative β-phase fraction $F\left(\beta \right)$ was calculated using Equation (1) [15]:

(1) $$F\left(\beta \right)=\frac{A_{\beta }}{\left(\frac{K_{\beta }}{K_{\alpha }}\right)A_{\alpha }+A_{\beta }}=\frac{A_{\beta }}{1.26A_{\alpha }+A_{\beta }}$$

where $A_{\alpha }\;$ and $A_{\beta }\;$ represent the absorbance values at 765 and 840 cm^−1^, corresponding to the α-phase and β-phase, respectively. $K_{\alpha }\;$and $K_{\beta }\;$ are the absorption coefficients of the α-phase and β-phase, with values of $6.1\times 10^{4}\;$ and $7.7\times 10^{4}\;$ cm^2^ mol^−1^, respectively.

**Figure 2:**
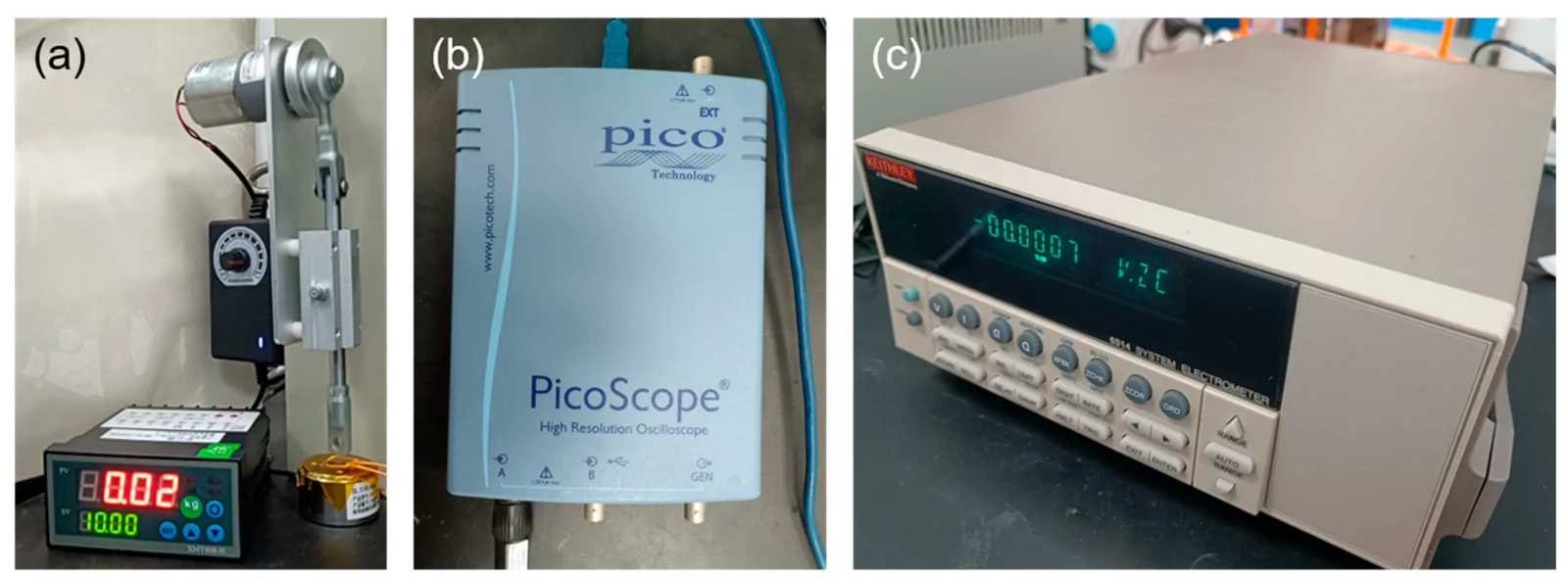
(**a**) Reciprocating motor. (**b**) PicoScope oscilloscope. (**c**) Keithley 6514 electrometer.

Electrical output measurements were conducted using a reciprocating motor (Model ZK-DIY-520, Shengda Machinery, Quanzhou, China) (Figure 2a) to provide periodic mechanical stimulation to the PVDF-HFP films. The motor operated at a frequency of approximately 1.42 Hz (85 rpm) and generated a cyclic compressive force of 10 N. The actuator periodically contacted the film surface at a position located 20 mm from the film edge. The open-circuit voltage (*V*_oc_) was recorded using a PicoScope oscilloscope (Model PicoScope 7, Pico Technology, St. Neots, UK) (Figure 2b), while the short-circuit current (*I*_sc_) was measured using a Keithley 6514 electrometer (Model 6514, Keithley Instruments Inc., Cleveland, OH, USA) (Figure 2c). All samples were tested under identical experimental conditions to ensure reliable comparison of their piezoelectric output performance.

## 3. Results and Discussion

### 3.1. Phase Analysis of PVDF-HFP Films

**Figure 3:**
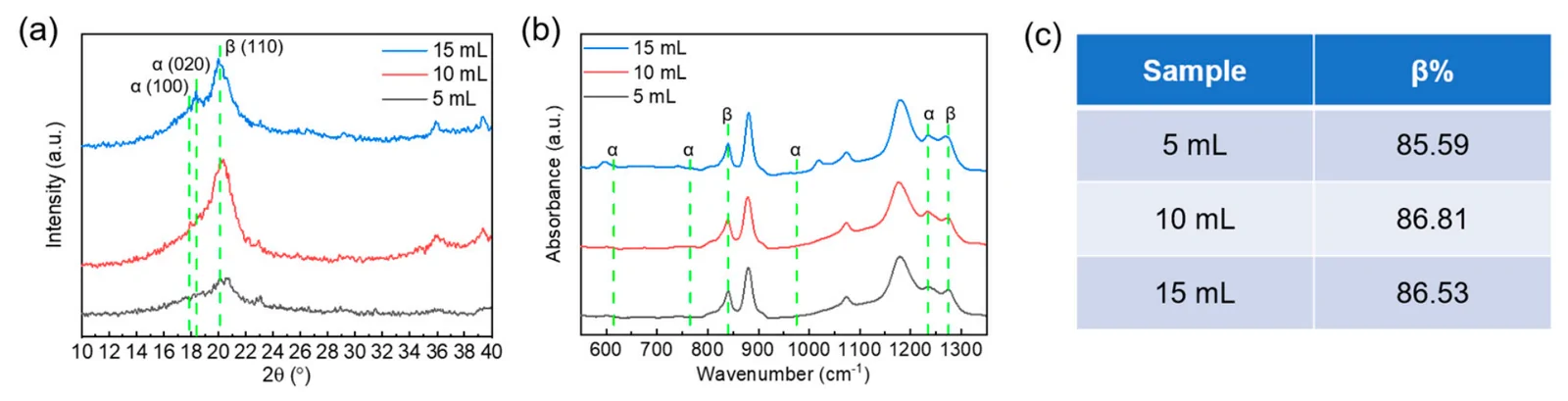
Structural characterization results of phase-separated PVDF-HFP films prepared with different spray volumes: (**a**) XRD patterns and (**b**) FTIR spectra of the 5 mL, 10 mL, and 15 mL samples. The characteristic diffraction peaks and absorption bands corresponding to the α and β phases are marked in the figures. (**c**) β-phase content of the samples.

Figure 3a presents the XRD patterns of phase-separated PVDF-HFP films prepared with different spraying volumes. All samples exhibit characteristic diffraction signals corresponding to the crystalline phases of PVDF-HFP. The diffraction peaks located at 17.9° and 18.4° are assigned to the nonpolar α-phase, while the broad diffraction peak centered at 20.4° corresponds to the electroactive β-phase [16]. Notably, the sample prepared with a spraying volume of 10 mL exhibits the most prominent β-phase diffraction peak, whereas both lower and higher spraying volumes result in relatively weakened β-phase signals. This result indicates that the formation of the polar crystalline phase is not simply proportional to the spraying volume, but instead depends on an appropriate phase separation window. When the spraying volume is insufficient, the nonsolvent-induced phase separation effect is limited, thereby restricting the rearrangement of molecular chains toward polar conformations. In contrast, excessive spraying leads to saturation, where the polar conformations no longer increase further, and may even induce overly rapid precipitation behavior, consequently suppressing the effective alignment of PVDF-HFP molecular chains into the β-phase.

The FTIR spectra shown in Figure 3b further confirm this non-monotonic crystal-phase evolution behavior [17,18]. The absorption peaks associated with the β-phase are most pronounced in the sample prepared with a spraying volume of 10 mL, while the α-phase-related signals remain more evident in the other groups. For each film, FTIR measurements were conducted at three different positions, and the β-phase content was calculated and averaged to reduce the measurement variation. As shown in Figure 3c, the calculated β-phase contents of the 5 mL, 10 mL, and 15 mL samples were 85.59%, 86.81%, and 86.53%, respectively. Since the β-phase is the primary electroactive crystalline phase responsible for the piezoelectric response of PVDF-HFP, the higher β-phase content suggests a greater potential for electromechanical conversion and sensing performance. This phenomenon may be attributed to the optimized phase separation conditions achieved at a moderate spraying volume. A moderate spraying volume results in differences in the β-phase content, as indicated by XRD and FTIR results. This variation may be associated with the changes in film formation conditions during solvent evaporation. Therefore, the 10 mL sample can be regarded as the optimal condition for promoting β-phase formation in phase-separated PVDF-HFP films. This optimized crystalline-phase composition may provide a structural basis for the subsequent flexible gait-sensing performance.

### 3.2. Electrical Output Response of PVDF-HFP Films

**Figure 4:**
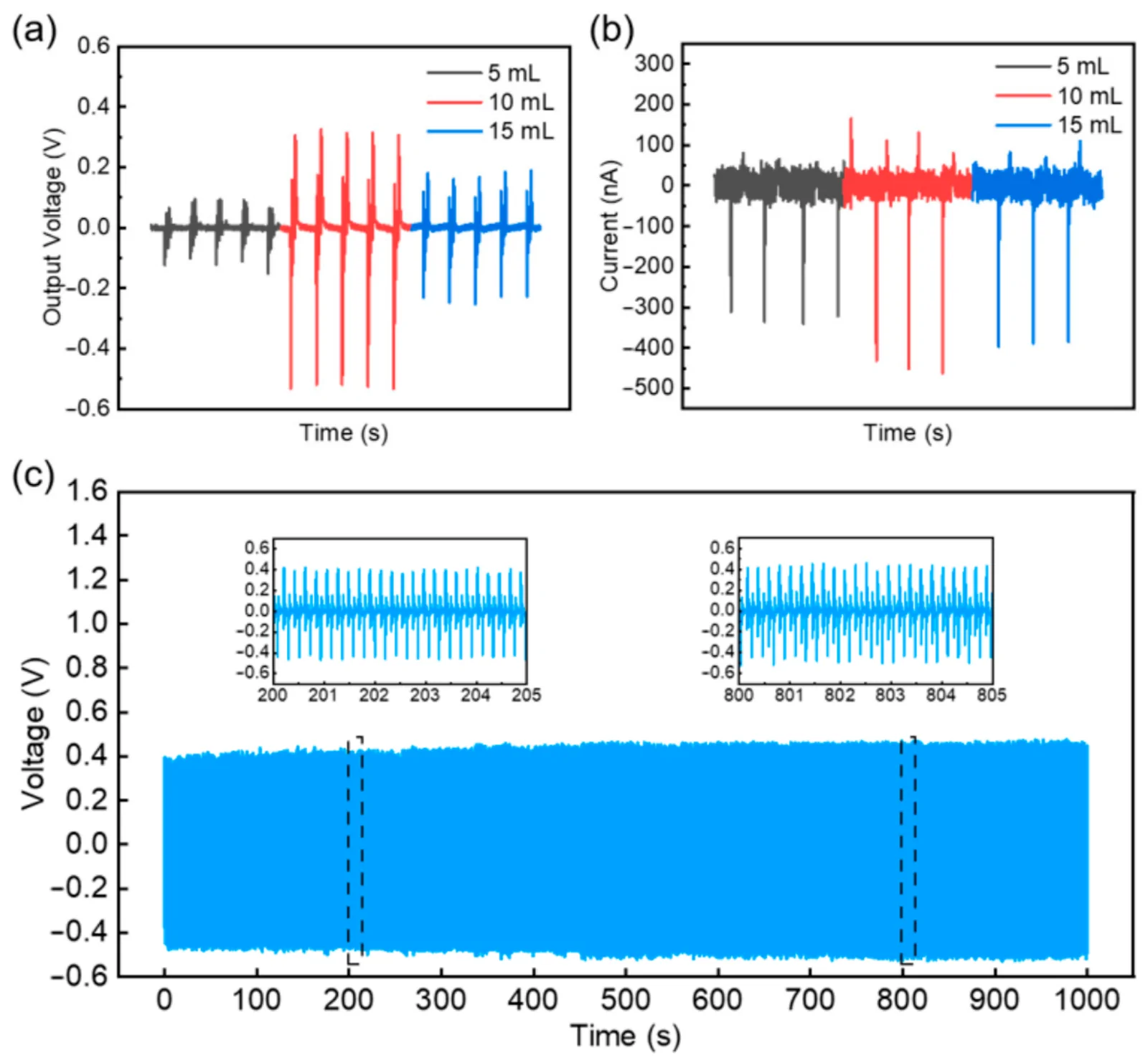
Electrical output performance of phase-separated PVDF-HFP films prepared with different spray volumes: (**a**) open-circuit voltage signals and (**b**) short-circuit current signals under periodic mechanical stimulation. (**c**) Continuous electrical output stability of the optimized 10 mL PVDF-HFP film in 1000 s, with enlarged views of the selected signal regions shown in the insets.

Figure 4 shows the electrical output responses of phase-separated PVDF-HFP films prepared with different spraying volumes under periodic mechanical stimulation. All samples exhibit stable and repeatable voltage and current signals, indicating that the fabricated films are capable of converting external mechanical deformation into electrical output. Significant differences in output intensity can be observed among the three groups. In particular, the sample prepared with a spraying volume of 10 mL exhibits the highest open-circuit voltage and short-circuit current, while both lower and higher spraying volumes result in relatively weaker electrical responses.

The enhanced electrical output observed for the 10 mL sample is consistent with its higher electroactive β-phase content obtained from the structural characterization results. In contrast, the other groups generate comparatively lower electrical signals, suggesting that the spraying volume plays an important role in determining the electrical response behavior of the phase-separated PVDF-HFP films.

To further investigate the operational stability of the phase-separated PVDF-HFP films, continuous electrical output measurements were performed for 1000 s under repeated mechanical stimulation. As shown in Figure 4c, the device maintained stable electrical output over approximately 1400 loading cycles without obvious signal attenuation or fluctuation drift. The consistent output behavior indicates high repeatability and short-term operational stability of the fabricated PVDF-HFP films during long-term mechanical deformation. These results further demonstrate the potential of the optimized PVDF-HFP films for flexible wearable sensing applications.

**Figure 5:**
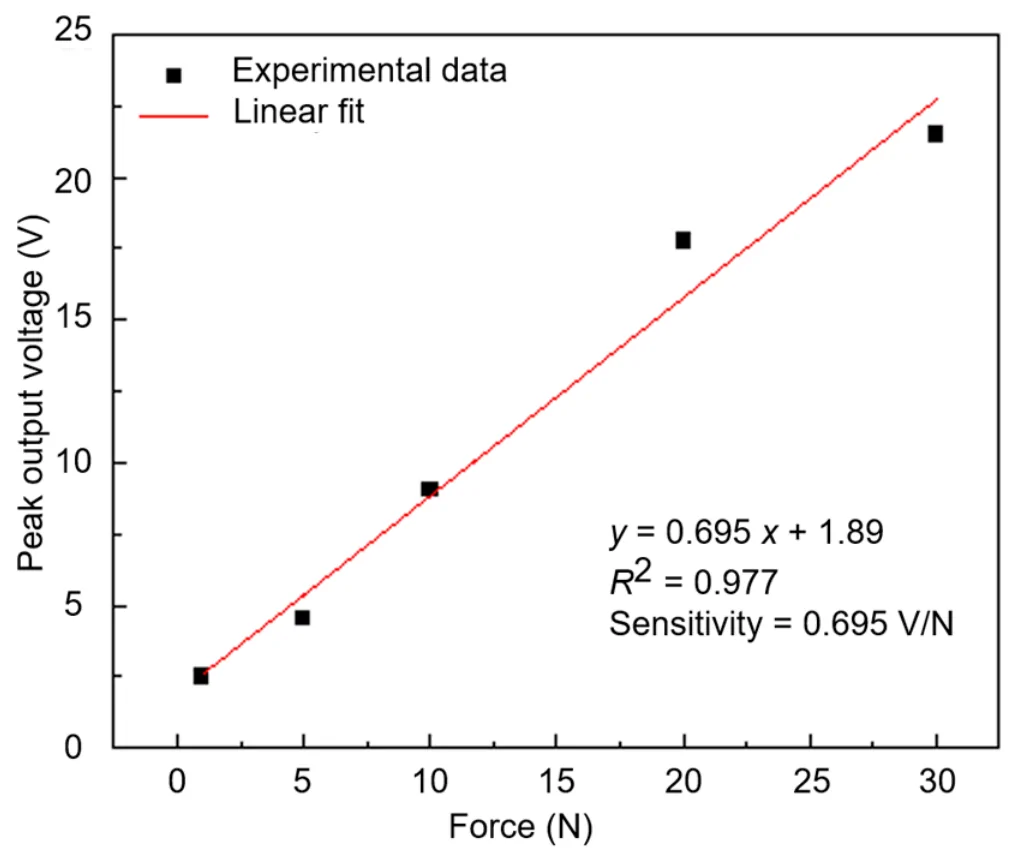
Peak output voltage of PVDF-HFP piezoelectric film under different external forces and the linear fitting curve.

To further evaluate the sensing performance of the optimized PVDF-HFP sensor, the relationship between the applied force and output voltage was investigated. As shown in Figure 5, the output voltage increased with increasing applied force over the range of 1–30 N. A linear fitting yielded a sensitivity of 0.695 V/N with a coefficient of determination (*R*^2^) of 0.997, indicating an excellent linear response to external force. The good linearity and stable force-dependent output demonstrate the capability of the sensor for quantitative force monitoring and wearable gait-sensing applications.

In Table 1, the sensitivity and measurement range of the proposed sensor with previously reported PVDF-HFP-based piezoelectric sensors are compared. Although some reported devices exhibit higher sensitivities, their effective operating ranges are relatively limited. In contrast, the phase-separated PVDF-HFP sensor developed in this work achieves a balance between sensitivity, linear response, and measurement range, which is advantageous for wearable gait-sensing applications, where the applied force varies considerably under different motion conditions.

### 3.3. Wearable Gait Signal Detection

Based on the structural characterization and electrical output analyses, the PVDF-HFP film prepared with a spraying volume of 10 mL was selected as the optimized sample for subsequent wearable sensing measurements. Compared with the other groups, the 10 mL sample exhibited the highest β-phase content together with the enhanced voltage and current output responses, indicating improved electromechanical conversion capability under mechanical deformation.

**Figure 6:**
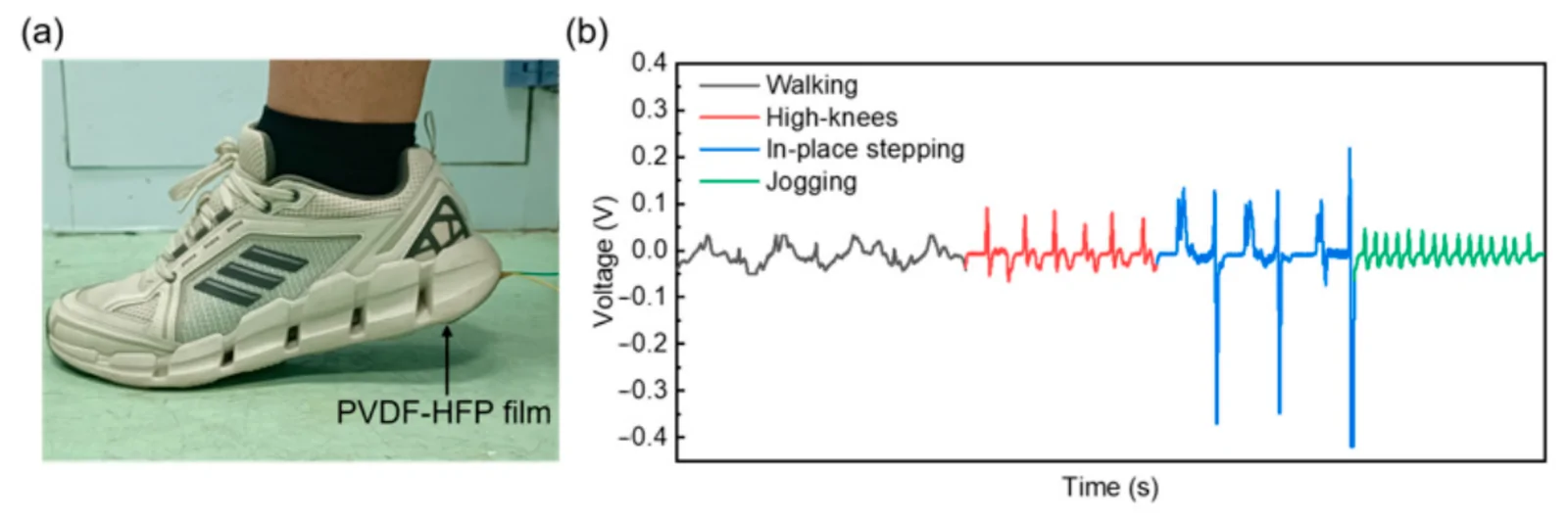
(**a**) The PVDF-HFP film attached to the shoe sole. (**b**) Wearable gait-sensing performance of the PVDF-HFP film under different human motion states, including walking, high-knees, in-place stepping, and jogging.

In this work, the wearable experiments were conducted using a single healthy adult male volunteer. The sensor was attached to the shoe insole (Figure 6a) to record signals under four representative motion states (walking, jogging, in-place stepping, and high-knee exercise). Multiple repeated trials were performed under consistent conditions to evaluate the feasibility of motion-state classification. Different motion behaviors generate distinguishable electrical response patterns. The walking signal exhibits relatively small and irregular fluctuations, corresponding to the moderate and variable pressure changes during normal gait. In comparison, the high-knees motion produces more obvious periodic peaks, indicating stronger and more regular mechanical stimulation applied to the sensor.

Notably, the stepping motion generates the largest electrical response, with pronounced positive and negative signal peaks, suggesting that this motion induces the strongest instantaneous mechanical deformation on the PVDF-HFP film. In contrast, the jogging signal exhibits the most stable and continuous periodic waveform, reflecting the repetitive and rhythmic characteristics of jogging motion. The distinguishable electrical signatures obtained from different motion states demonstrate that the optimized phase-separated PVDF-HFP film possesses effective gait-monitoring capability and may provide a reliable signal basis for subsequent machine learning-based motion recognition.

### 3.4. Machine Learning-Based Motion Classification

To further evaluate the distinguishability of gait-related electrical signals generated by the optimized PVDF-HFP film, machine learning-based motion recognition was performed using four commonly used classification models, including Logistic regression (LR) [22], Support vector machine (SVM) [23,24], Extreme gradient boosting (XGBoost) [25], and Multilayer perceptron (MLP). These models were selected due to their respective advantages in linear classification, small-sample recognition, nonlinear feature learning, and neural network-based pattern analysis [26]. The electrical signals collected from different motion states, including walking, high-knees, in-place stepping, and jogging, were used as the input dataset for model training and testing. To ensure consistency, all signals were acquired using the same sensor structure, testing circuit, and acquisition conditions as those used in the previous electrical-output measurements.

**Figure 7:**
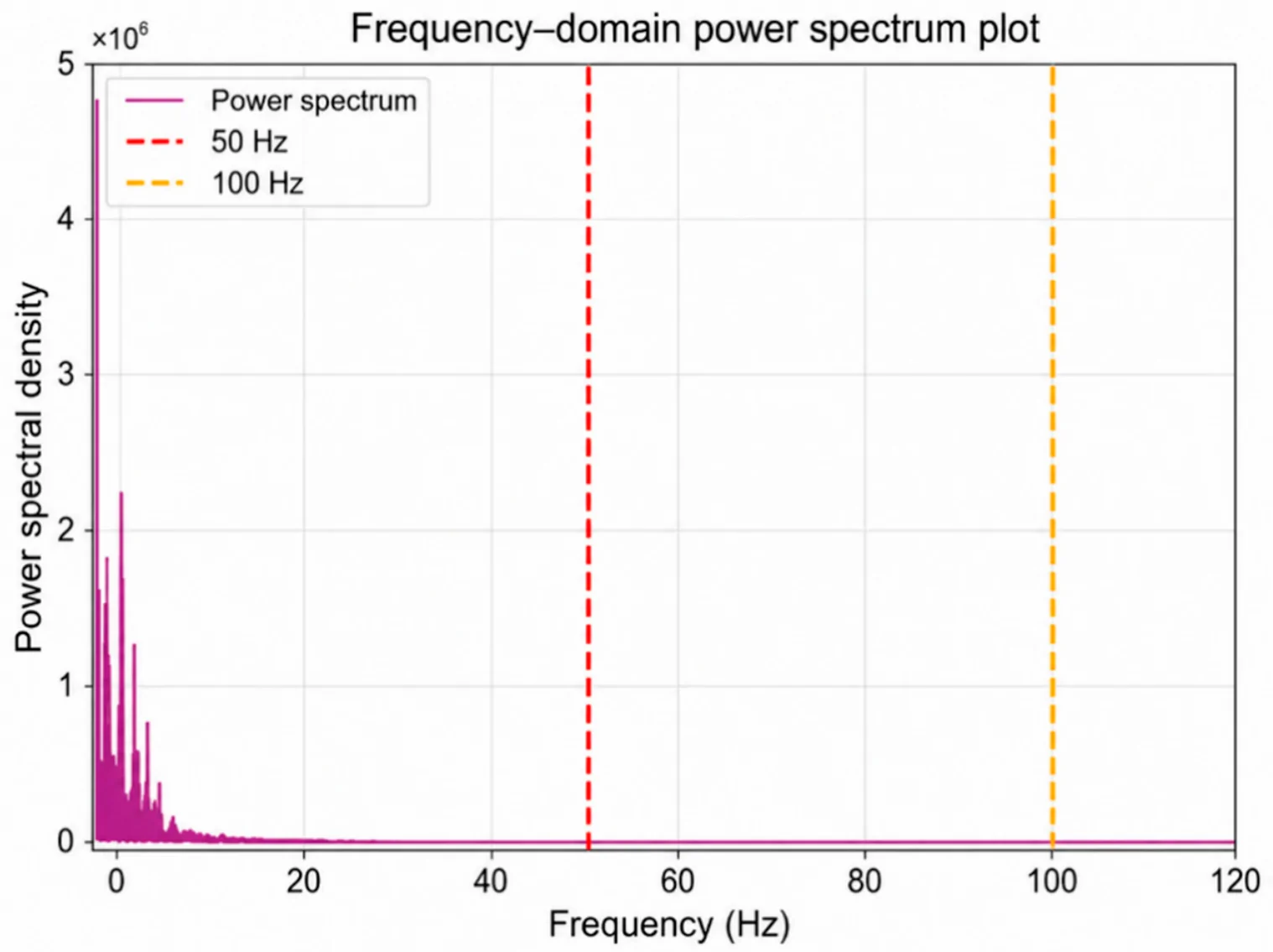
Frequency-domain power spectral density analysis of the collected gait signals.

Before machine learning analysis, the unstable initiation and termination regions were removed to reduce motion transition artifacts and focus on the steady-state characteristics of each gait pattern. Frequency-domain analysis was then performed using power spectral density analysis, as shown in Figure 7. The signal energy was mainly concentrated in the low-frequency region below 10 Hz, which is consistent with the typical frequency range of human gait motion. In addition, no obvious spectral peaks were observed at 50 Hz or 100 Hz, indicating that the hardware system effectively suppressed power-frequency interference and high-frequency noise during signal acquisition. Therefore, no additional software filtering was applied in this study, and the screened raw signals were directly used for subsequent feature extraction and machine learning modeling in order to preserve the original motion-related signal characteristics.

The extracted sensing signals were preprocessed and transformed into feature vectors prior to classification. Different machine learning algorithms employed their respective feature selection and optimization strategies according to their implementation characteristics. The purpose of this study was to evaluate the feasibility of distinguishing different motion states using signals generated by the proposed PVDF-HFP sensor, rather than to establish an optimized machine learning benchmark.

**Figure 8:**
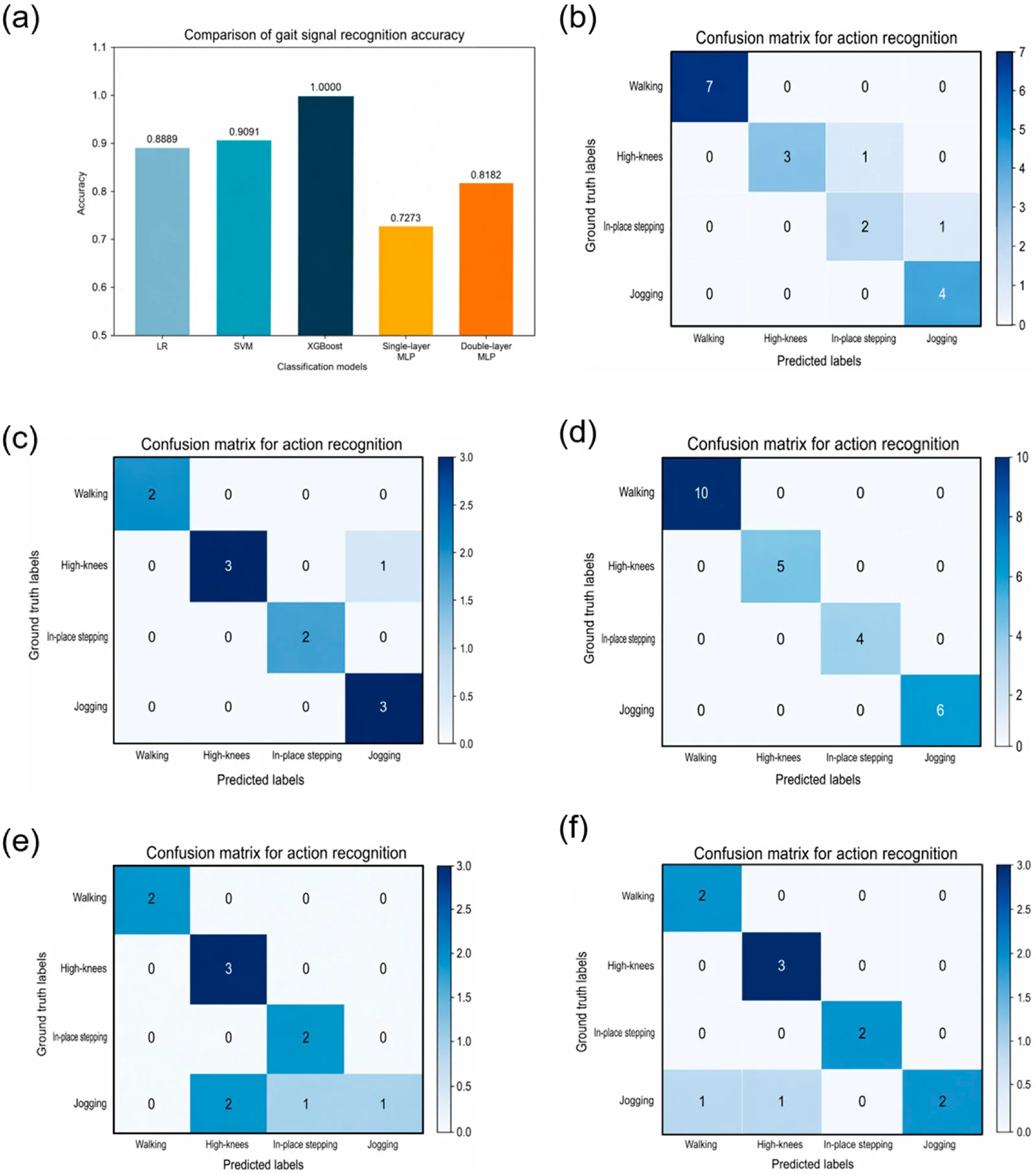
Machine learning-based motion recognition results of the collected gait signals: (**a**) comparison of classification accuracies for different machine learning models; confusion matrices of (**b**) LR, (**c**) SVM, (**d**) XGBoost, (**e**) single-layer MLP, and (**f**) double-layer MLP models.

The classification accuracies of different machine learning models are summarized in Figure 8a, while the corresponding confusion matrices are shown in Figure 8b–f. Overall, all classification models demonstrate effective recognition capability for the four motion states, indicating that the gait-related electrical signals generated by the optimized PVDF-HFP sensor possess distinguishable signal features suitable for motion classification.

Among the tested models, the LR model achieves an accuracy of 88.89%, demonstrating relatively stable classification performance with most motion states successfully recognized. However, a small degree of confusion can still be observed between high-knees and in-place stepping, as well as between in-place stepping and jogging. The SVM model further improves the classification performance, achieving an accuracy of 90.91%, with only minor misclassification observed in the high-knees category. These results demonstrate the effectiveness of traditional machine learning models for distinguishing gait-related signal patterns under limited sample conditions.

Compared with the traditional linear and kernel-based models, the XGBoost model exhibits the best overall recognition performance, achieving a classification accuracy of 100%. As shown in the confusion matrix, all motion categories are correctly identified without obvious misclassification, indicating that XGBoost achieved the highest accuracy on the current dataset, suggesting that the extracted signal features contain discriminative information for motion state classification. This result further suggests that the generated sensing signals contain sufficiently distinguishable dynamic characteristics for accurate motion recognition. Although XGBoost achieved accuracy of 100% on the current dataset, we acknowledge that the dataset was collected from a single participant and is therefore limited in size. Accordingly, the reported results should be considered as a preliminary proof-of-concept demonstration rather than evidence of generalized recognition capability.

For the MLP models, the single-layer and double-layer structures achieve classification accuracies of 72.73% and 81.82%, respectively. Although both MLP models successfully recognize most motion states, relatively higher confusion is observed in the jogging category compared with XGBoost and SVM. In particular, jogging signals are partially misclassified as high-knees or in-place stepping, possibly because these motions share similar rhythmic and periodic signal characteristics. In addition, the relatively limited dataset size may restrict the learning efficiency of neural network-based models. Nevertheless, the MLP models still maintain acceptable recognition capability, indicating that the collected gait signals possess sufficient feature information for neural network-based classification.

To provide a more comprehensive evaluation of classification performance, precision, recall, and F1-score were additionally calculated and are summarized in Table 2. These metrics complement the accuracy results by providing a more detailed assessment of classification behavior.

Overall, the machine learning results demonstrate that the optimized phase-separated PVDF-HFP wearable sensor can effectively capture distinguishable gait-related electrical signals under different human motion states. The successful motion classification further highlights the potential applications of the fabricated flexible sensor in wearable motion monitoring, gait recognition, and human motion interaction systems.

## 4. Conclusions

In this work, a flexible phase-separated PVDF-HFP film was successfully fabricated and applied to wearable gait sensing and machine learning-assisted motion recognition. By regulating the loading masses of PVDF-HFP during the phase separation process, the crystalline-phase evolution and electrical output behavior of the PVDF-HFP films were systematically investigated. Structural characterization results from XRD and FTIR analyses demonstrated that the film prepared with a spraying volume of 10 mL exhibited the highest electroactive β-phase content, reaching 86.81%, indicating that an appropriate phase separation condition plays an important role in promoting the formation of polar crystalline structures.

Electrical output measurements further revealed that the optimized 10 mL sample generated enhanced open-circuit voltage and short-circuit current responses under periodic mechanical stimulation, together with stable electrical output during continuous testing. Based on its superior structure and electrical performance, the optimized PVDF-HFP film was subsequently employed for wearable gait sensing. Different human motion states, including walking, high-knees, in-place stepping, and jogging, produced distinguishable electrical signal patterns, demonstrating the capability of the fabricated flexible sensor for motion monitoring applications.

To further evaluate the distinguishability of the collected gait signals, multiple machine learning models, including LR, SVM, XGBoost, and MLP, were applied for motion classification. The classification results demonstrated effective recognition performance for different gait-related motion states, with XGBoost exhibiting the strongest overall classification capability. These results suggest the feasibility of integrating the proposed sensor with machine learning methods for motion state analysis.

## Figures and Tables

**Table 1:** Comparison of sensitivity and measurement range of representative PVDF-HFP-based piezoelectric sensors.

Material	Fabrication	Sensitivity (V/N)	Range (N)
PDMS/PVDF-HFP [19]	Electrospinning	0.68	10–70
25 wt.% Vermiculite/ PVDF-HFP [16]	Electrospinning	0.02857	5–40
PVDF-HFP/ 1 wt.% CNC Nanofibers [20]	Electrospinning	0.146	1–30
MOF@MoS_2_/PVDF-HFP [21]	Electrospinning	1.83	0–2
PVDF-HFP (This Work)	Phase Separation	0.695	1–30

**Table 2:** Performance comparison of different classifiers.

Model	Accuracy (%)	Precision (%)	Recall (%)	F1-Score (%)
LR	88.89	86.67	85.42	85.32
SVM	90.91	93.75	93.75	92.86
XGBoost	100.00	100.00	100.00	100.00
Single-layer MLP	72.73	81.67	81.25	73.75
Double-layer MLP	81.82	85.42	87.50	83.10

## Data Availability

The raw/processed data required to reproduce these findings cannot be shared at this time as the data also forms part of an ongoing study.
